# Mammary collagen architecture and its association with mammographic density and lesion severity among women undergoing image-guided breast biopsy

**DOI:** 10.1186/s13058-021-01482-z

**Published:** 2021-11-09

**Authors:** Clara Bodelon, Maeve Mullooly, Ruth M. Pfeiffer, Shaoqi Fan, Mustapha Abubakar, Petra Lenz, Pamela M. Vacek, Donald L. Weaver, Sally D. Herschorn, Jason M. Johnson, Brian L. Sprague, Stephen Hewitt, John Shepherd, Serghei Malkov, Patricia J. Keely, Kevin W. Eliceiri, Mark E. Sherman, Matthew W. Conklin, Gretchen L. Gierach

**Affiliations:** 1 Division of Cancer Epidemiology and Genetics, National Cancer Institute, 9609 Medical Center Dr., Rm 7-E238, Bethesda, MD 20892 USA; 2 Division of Population Health Sciences, Royal College of Surgeons in Ireland, Dublin, Ireland; 3 University of Vermont College of Medicine and Vermont Cancer Center, Burlington, VT USA; 4 The University of Texas MD Anderson Cancer Center, Houston, TX USA; 5 University of Hawaii Cancer Center, Honolulu, HI USA; 6 Applied Materials, Santa Clara, CA USA; 7 Department of Cell and Regenerative Biology and Carbone Cancer Center, School of Medicine and Public Health, University of Wisconsin-Madison, 1111 Highland Ave., WIMR II Rm. 4528, Madison, WI 53705 USA; 8 Department of Biomedical Engineering, University of Wisconsin-Madison, Madison, WI USA; 9 Mayo Clinic, Jacksonville, FL USA

**Keywords:** Collagen fibers, Mammographic density, Breast pathology, Second harmonic generation imaging, Breast diseases, Breast neoplasms

## Abstract

**Background:**

Elevated mammographic breast density is a strong breast cancer risk factor with poorly understood etiology. Increased deposition of collagen, one of the main fibrous proteins present in breast stroma, has been associated with increased mammographic density. Collagen fiber architecture has been linked to poor outcomes in breast cancer. However, relationships of quantitative collagen fiber features assessed in diagnostic biopsies with mammographic density and lesion severity are not well-established.

**Methods:**

Clinically indicated breast biopsies from 65 in situ or invasive breast cancer cases and 73 frequency matched-controls with a benign biopsy result were used to measure collagen fiber features (length, straightness, width, alignment, orientation and density (fibers/µm^2^)) using second harmonic generation microscopy in up to three regions of interest (ROIs) per biopsy: normal, benign breast disease, and cancer. Local and global mammographic density volumes were quantified in the ipsilateral breast in pre-biopsy full-field digital mammograms. Associations of fibrillar collagen features with mammographic density and severity of biopsy diagnosis were evaluated using generalized estimating equation models with an independent correlation structure to account for multiple ROIs within each biopsy section.

**Results:**

Collagen fiber density was positively associated with the proportion of stroma on the biopsy slide (*p* < 0.001) and with local percent mammographic density volume at both the biopsy target (*p* = 0.035) and within a 2 mm perilesional ring (*p* = 0.02), but not with global mammographic density measures. As severity of the breast biopsy diagnosis increased at the ROI level, collagen fibers tended to be less dense, shorter, straighter, thinner, and more aligned with one another (*p* < 0.05).

**Conclusions:**

Collagen fiber density was positively associated with local, but not global, mammographic density, suggesting that collagen microarchitecture may not translate into macroscopic mammographic features. However, collagen fiber features may be markers of cancer risk and/or progression among women referred for biopsy based on abnormal breast imaging.

**Supplementary Information:**

The online version contains supplementary material available at 10.1186/s13058-021-01482-z.

## Background

Mammographic density is a radiological reflection of breast fibroglandular content, which histologically corresponds to the quantity of epithelium and stroma [1]. Epidemiologic investigations have established that increased mammographic density is a strong breast cancer risk factor [2], but mechanisms that mediate underlying risk are poorly understood [1]. Environmental and biological factors are thought to be responsible for variations in breast tissue composition that are reflected in inter-individual differences in mammographic density [3]. However, clinically indicated biopsies of women with high mammographic density vary with regard to severity of biopsy diagnosis and epithelial–stromal content [4], and most women with high mammographic density do not develop cancer. Therefore, there is an important clinical gap of identifying women with high mammographic density who are more likely to develop breast cancer.

The mammary extracellular matrix (ECM) is the non-cellular component of the stroma that provides essential physical scaffolding and initiates crucial biochemical and biomechanical processes required for tissue development, differentiation, and homeostasis, and contributes importantly to carcinogenesis [5]. Collagen is one of the main fibrous proteins of the ECM. The relationship between histologic measures of collagen organization and radiologic mammographic density is not well understood. Several studies have found that greater collagen deposition in breast tissues derived from autopsies and biopsies is associated with increased percent mammographic density [6–10]. These studies primarily assessed relationships of collagen deposition with global measures of percent mammographic dense area [6, 7, 9, 10], and one study used X-rays of breast tissue slices [8]. Apart from collagen deposition, relationships between other collagen fiber features and mammographic density are not well established. Small studies have found that greater collagen alignment, and hence increased tissue stiffening, are features that may be related to breast cancer risk [5], and that higher collagen density and thicker collagen fibers were associated with higher global percent mammographic dense area [9–11]. In addition to global measures, localized measures of mammographic density in well-defined regions of interest may help to further our understanding of relationships between stromal collagen microstructure organization and radiologic features indicative of increased breast cancer risk.

To investigate relationships of collagen content and its organizational features with global and local volumetric mammographic density measures, we examined diagnostic breast biopsies using second harmonic generation (SHG) imaging, which is a high-resolution, label-free imaging technique that allows direct visualization of individual collagen in fibers routinely prepared, hematoxylin and eosin (H&E)-stained slides. SHG facilitates not only the quantification of the amount of collagen, but also the extraction of individual collagen fiber characteristics such as length, straightness, width, density, and alignment. Since collagen has also been suggested in animal models to be involved in the early stages of breast carcinogenesis [12], and may be an indicator of subsequent malignant transformation [13], we also explored associations between collagen fiber features and severity of breast biopsy diagnoses. In addition, three tumor-associated collagen signatures (TACS) have been previously defined [14, 15], including TACS-1 defined by a region of dense collagen, TACS-2 defined by straightened collagen fibers and TACS-3 defined by collagen fibers that are perpendicularly aligned to the tumor boundary. TACS-3 has been found to facilitate breast cancer invasion. We therefore explored whether TACS-3, as visually scored by study pathologists, was also associated with malignant transformation.

## Materials and methods

### Study population

The National Cancer Institute (NCI) Breast Radiology Evaluation and Study of Tissues (BREAST) Stamp Project is a cross-sectional epidemiologic study of mammographic density undertaken at the University of Vermont College of Medicine and the University of Vermont Medical Center, as previously described [16]. Briefly, 465 women, aged 40–65 years, who were referred for an image-guided breast biopsy (2007–2010) were enrolled: eligible women had not had breast cancer or breast surgery within the preceding year, did not have breast implants, and were not taking breast cancer chemoprevention. Study participants completed a questionnaire and a follow-up telephone interview. Participants underwent clinically indicated ultrasound-guided (14-gauge needle) or vacuum-assisted (9-gauge needle) breast biopsies, which were processed as formalin-fixed paraffin-embedded blocks, sectioned at 5 μm thickness, H&E-stained and collected for research. Participants provided written informed consent in accordance with approvals from the NCI Special Studies Institutional Review Board (IRB) and the University of Vermont IRB.

### Analytical population

This current study used a matched case–control design. Pathological diagnoses from biopsy pathology reports were used to determine case–control status for the breast cancer cases and benign breast disease (BBD) controls. Participants were excluded from the case–control selection if they did not undergo a radiologically guided breast biopsy (*N* = 12), did not have tissue collected (*N* = 1), went straight to surgery (*N* = 2), did not have ipsilateral breast density measurements available (*N* = 44) or did not have a H&E-stained biopsy available for investigation of collagen assessment (*N* = 1). Of the remaining participants eligible available for selection, all women who received a biopsy diagnosis of either in situ (*n* = 32) or invasive (*n* = 33) breast cancer were selected as cases (*n* = 65). The remaining available eligible women diagnosed with BBD were considered as potential controls. Controls were selected by randomly matching to cases on age (5-year age groups), body mass index (BMI) and menopausal status. Eight additional controls whose slides were used for a pilot of this project were also included for a total of 73 controls and 65 cases in the analytic population.

### Mammographic density assessment and histologic tissue composition metrics

Volumetric density assessment was performed using a Single X-ray Absorptiometry (SXA) breast density phantom [17]. Quantitative global [16] and localized [18] measures of dense fibroglandular tissue volume (FGV, cm^3^) and percent fibroglandular tissue volume (% FGV) were assessed in pre-biopsy craniocaudal views of the ipsilateral breast of the primary pathologic diagnosis and taken closest in time before breast biopsy. For localized assessment of perilesional % FGV measurements, the biopsy location and lesion radius were identified on pre-biopsy mammograms by the study radiologist [18]. Localized FVG and % FVG measurements at the biopsy target and a volume ~ 0-2mm^3^ surrounding but excluding the biopsy target location were included in this analysis.

H&E-stained tissue sections from each diagnostic breast biopsy were digitized at 20X magnification (Aperio ScanScope CS). An image-based algorithm based on convolutional neural networks was applied to digitized whole slide biopsy images (WSI) to quantify areas of epithelium, stroma and adipose tissues [19, 20], and their proportions were computed by dividing by total tissue area on the slide.

### Selection of regions of interests (ROIs)

**Fig. 1:**
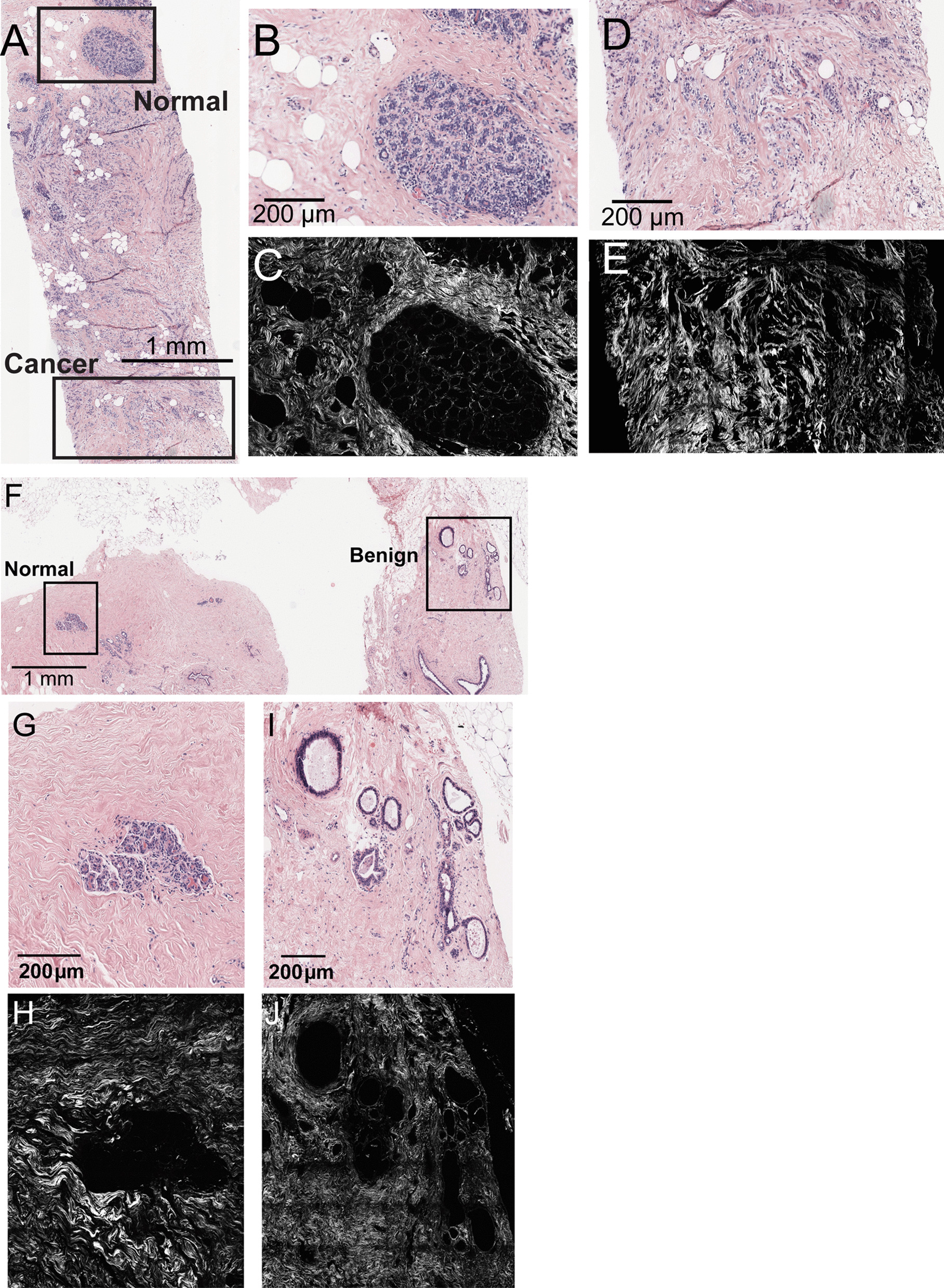
Collagen fibers were measured using second harmonic generation (SHG) microscopy in each of the selected regions of interest (ROIs) of H&E-stained WSI from diagnostic breast biopsy sections. Panel **a** illustrates ROI selection for a breast cancer case, with magnifications of the normal **b** and cancer **d** ROIs, and their corresponding SHG images of collagen fibers shown in Panels **c** and **e.** Panel **f** illustrates ROI selection for a benign breast disease control, with magnifications of the normal **g** and benign **i** ROIs, and their corresponding SHG images of collagen fibers shown in Panels **h** and **j**

Up to three regions of interest (ROI) in each H&E-stained WSI were selected by a pathologist (MES) for collagen fiber measurement and classified as follows: *normal* (normal lobules or ducts), *benign* (sclerotic/atrophied lobules or ducts; non-proliferative BBD; other discrete non-proliferative benign breast diagnoses; ductal hyperplasia without atypia and atypical ductal or lobular hyperplasia; sclerosing adenosis) or *cancer* (*in-situ* or invasive carcinoma). In benign breast biopsies, we identified ROIs containing normal and benign findings; in biopsies from cancer patients, we identified ROIs with normal, benign and cancer findings (Fig. 1). Cancer ROIs were not always present in the WSI of cancer cases.

### Collagen fiber assessment in diagnostic breast biopsies

For assessment of collagen, H&E-stained tissue sections were imaged with a previously described custom-built integrated SHG/bright-field imaging system [21]. A MIRA 900 Ti:Sapphire laser tuned to 780 nm excitation was utilized with a 40X/1.25 NA water immersion objective lens (Nikon, Melville, NY). SHG light was collected in the forward direction with a 0.54 NA condenser lens (ThorLabs), a 390/22 nm bandpass filter (Semrock) and a H7422-40P GaAsP photomultiplier detector. Timing between the galvanometer scanners, signal acquisition, and motorized stage positioning was achieved using our custom software called WiscScan (https://eliceirilab.org/software/wiscscan/). Bright-field images were captured with the same system using a MCWHL2 white LED lamp (ThorLabs) set up for Kohler illumination. White light from this lamp was separated from SHG light traveling through the condenser assembly using a short pass dichroic mirror with a cutoff at 670 nm (Semrock). A red–green–blue (RGB) camera (QImaging, Surrey, BC, Canada) was used to capture bright-field images through WiscScan to allow for acquisition within a single application. Prior to SHG imaging, the H&E-stained slide was scanned in bright-field mode to navigate to the annotated ROIs. SHG images were captured as a z-stack of 3 images spaced 3 μm apart, and then maximum-intensity projected to capture the entire axial field of view. Individual images of 1024 × 1024 pixels were captured using an electronic zoom of 3, resulting in an image size of 180 μm^2^. To image the entire area of interest, an array of multiple SHG images was acquired in a tiled fashion with 5% overlap between images using automation provided by WiscScan. Stage positions for individual images and pixel size data were stored [22] and this was then used by the Grid/Collection stitching ImageJ plugin [23] to reassemble a high-resolution large field view of the imaged area (approximately 1 mm^2^, but varying from location to location).

Two different custom-written open-source software packages, CT-FIRE and CurveAlign [24, 25] were used to analyze collagen fiber organization in SHG images. Both programs execute a curvelet transform of the SHG image [26]. Each curvelet had an x–y image location and orientation. The CT-FIRE program merged unitary curvelets into a single extracted fiber which recapitulated the collagen fiber. We also determined the total length (following the contour of the fiber), end-to-end length (i.e., straight distance between one end of the fiber to the other end), and width of each individual collagen fiber in the SHG image (Fig. 1). The ratio of end-to-length to total length was computed as a measure of straightness (serpentine appearance of fibers). CurveAlign measured fiber alignment (anisotropy) as a function of fibers within a pre-defined box of size 44.91 μm × 44.91 μm. Each individual image in the array was analyzed and the data combined. For orientation data, a boundary separating the collagen matrix from breast epithelial cells was created in CurveAlign using the stitched image, which was then used to measure fiber angle with respect to that boundary for each individual fiber. Stitched images were only available for a subset of women (38 cases and 44 controls).

With regard to TACS-3 [15], three reviewers (MA, PL and MWC) independently scored each ROI for the presence of TACS-3. For ROIs with discordant scoring by at least one of the three reviewers (*N* = 51), the three reviewers rescored the ROI a second time. The final score for the presence/absence of TACS-3 was defined as the score given by at least two reviewers following rescoring.

### Statistical analysis

Statistical differences in participant characteristics by case–control status were calculated using a Fisher exact test for categorical variables and a t-test for continuous measures. Collagen fiber characteristics examined included: (1) length, with higher values indicating longer fibers and lower values indicating shorter fibers; (2) straightness, ranging between 0–100, with higher values indicating straighter fibers and lower values indicating curvier fibers; (3) width, with higher values indicating thicker fibers and lower values indicating thinner fibers; (4) alignment, ranging between 0 and 100, with higher values indicating greater isotropic fiber alignment and lower values indicating fibers are more randomly ordered; (5) density, computed as the total number of fibers per 100 µm^2^; and (6) orientation, which measures collagen fiber angle with respect to the boundary surrounding the epithelial cells in the ROI and ranges between 0° and 90°. Collagen fibers characteristics, except for density, were estimated as either the average (for length, width, and number of fibers) or the median (for straightness, alignment, and orientation) at each ROI to avoid analyses being driven by outliers. These average/median values were used in all subsequent analyses. Whether we analyzed the average or median for each of the fiber characteristics was decided a priori considering for each characteristic whether the average or the median was biological meaningful.

Associations were first evaluated between collagen fiber features and participant characteristics using generalized estimating equation (GEE) linear models with an independent correlation structure to account for within-woman correlations for the different ROIs within an H&E-stained slide. The outcome of these models was the fiber characteristic. Other variables included in the model were the diagnosis of the ROI and the case–control status of the woman. Second, associations of histologic measures of breast tissue composition and continuous mammographic density measurements (global or localized) with collagen characteristics were evaluated using GEE models with an independent correlation structure, with the tissue composition or the mammographic density measure being the outcome, and collagen fiber characteristics the independent variables. Quantitative tissue composition and volumetric mammographic density measures were transformed by taking the square root to better approximate a normal distribution. Models also included variables for ROI diagnosis and case–control status of the woman. Beta coefficients (β) of all GEE models indicated the average change of the outcome variable per unit change of the independent variable. Because the units for different fiber characteristics vary widely (e.g., absolute scale for fiber length in μm *vs* values between 0 and 100 for straightness), we standardized to 1 standard deviation (SD) of each collagen feature. Analyses were done overall and stratified by case–control status as indicated. Finally, we evaluated associations between collagen fiber characteristic and severity of the ROI diagnosis or the overall diagnosis of the women using GEE logistic models with an independent correlation structure to estimate odds ratios (ORs) and corresponding 95% confidence intervals (CIs). Models evaluating collagen fiber associations with the severity of ROI diagnoses included a variable for case–control status of the woman.

*P *values were two-sided, and P ≤ 0.05 was considered statistically significant. All analyses were performed using the R software environment (version 3.0.2).

## Results

### Characteristics of the study population

Of the 138 women included in this analysis, 65 had an invasive or in situ cancer diagnosis and 73 had a BBD diagnosis. Matching factors, such as age (mean (SD) age of cases: 52.8 (6.2) years; mean (SD) age of controls: 51.9 (6.1) years), BMI (< 25 kg/m^2^: 47.7% of cases and 45.2% of controls) and postmenopausal status (53.8% of cases and 52.1% of controls) were similar between cases and controls (Additional file 1: Table S1). Compared with controls, cases were more likely to have a first birth after age 30 and, as expected, had larger lesions identified on pre-biopsy mammograms. Among the cases, tumors tended to be small (78% were < 2 cm; Additional file 1: Table S2) and ER or PR positive (> 74%).

### Distribution of collagen fiber features by biopsy diagnosis and selected ROIs on biopsy sections

There were 243 ROIs identified and analyzed for this study (Additional file 1: Table S3), including 133 ROIs among the BBD controls and 110 ROIs among the cases. Most women had at least two ROIs identified and analyzed on each WSI. Of the 133 ROIs identified in the BBD controls, 71 (53.4%) ROIs had benign diagnoses and 62 ROIs (46.6%) represented the normal background tissue (Additional file 1: Table S3). Of the 110 ROIs identified in the cases, 56 ROIs (50.9%) had a cancer diagnosis, 39 (35.5%) had a benign ROI, and 15 ROIs (13.6%) represented the normal background tissue.

**Fig. 2:**
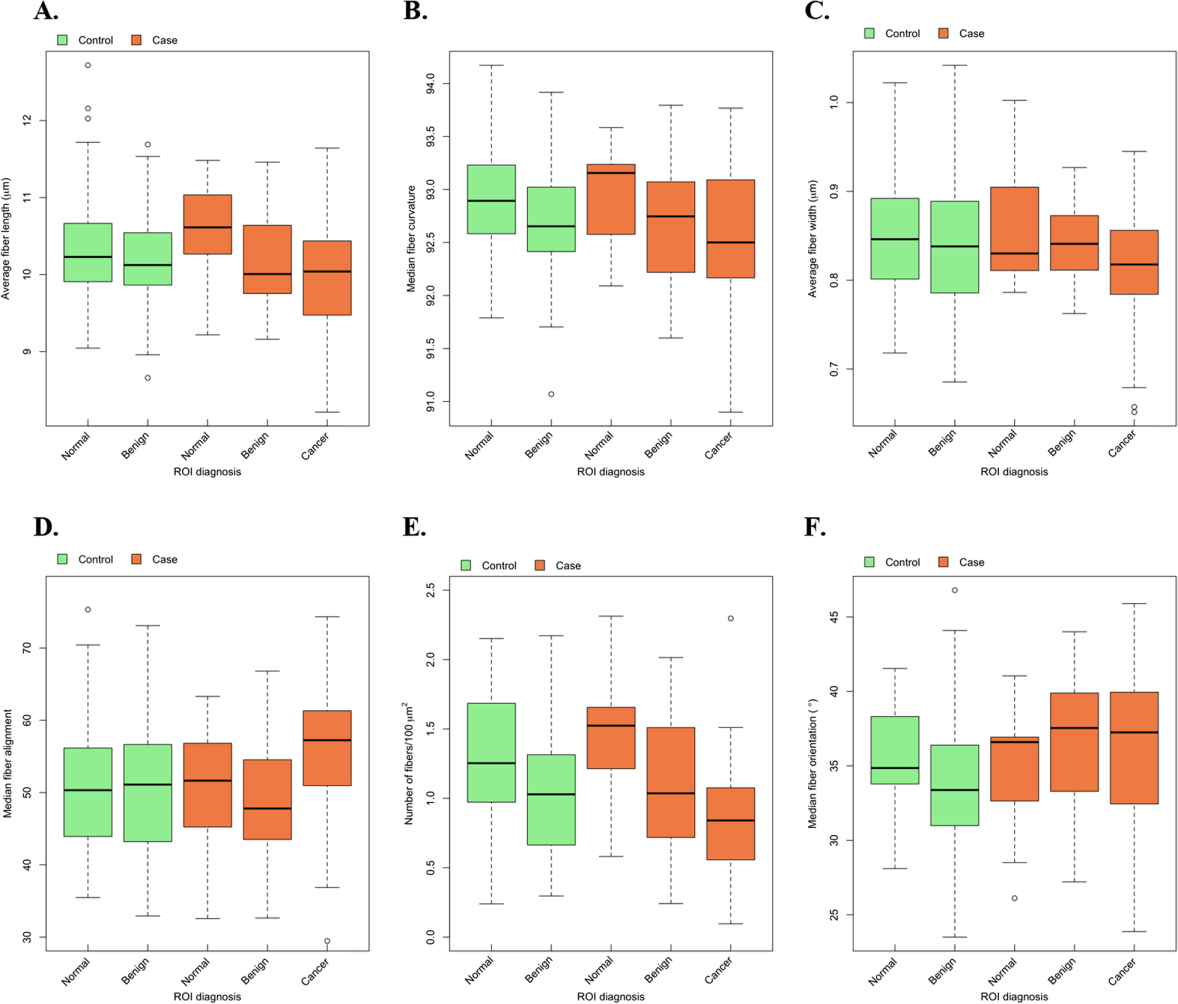
Boxplots of collagen fiber features among patients with a clinically indicated diagnostic breast biopsy (65 breast cancer cases and 73 controls) by overall biopsy diagnosis and within the normal, benign and malignant regions of interest (ROI) on H&E-stained breast biopsy sections. **a** Average fiber length: higher values indicate longer fibers. **b** Median fiber straightness (values between 0 and 100): higher values indicate straighter fibers, while lower values indicate curvier fibers. **c.** Average fiber width: higher values indicate thicker fibers, while lower values indicate thinner fibers. **d** Median fiber alignment (values between 0 and 100): higher values indicate greater isotropic alignment of fibers, while lower values indicate fibers are more randomly ordered. **e** Fiber density: higher values indicate greater number of fibers per 100 μm^2^. **f** Median fiber orientation (values between 0° and 90°): measures the angle of the collagen fiber with respect to the boundary surrounding the epithelial cells in the ROI

Approximately 3 million collagen fibers were included in this analysis. Among controls, median fiber number in normal ROIs was 7,894 and in benign ROIs was 11,106. For cases, the median fiber number was 9,836 in normal ROIs, 7,164 in benign ROIs and 11,552 in cancer ROIs. Distributions of collagen fiber features (length, straightness, width, alignment, density, and orientation) in different ROIs are shown in Fig. 2. In descriptive, unadjusted analyses, average fiber length, straightness, and density (# fibers/100 µm^2^) were greater in normal ROIs for both cases and controls, followed by benign ROIs, followed by cancer ROIs in the cancer cases (for formal statistical testing, see section “*Relation of collagen fiber characteristics with severity of ROI and biopsy diagnoses*” and Table 2).

### Relation of collagen fiber characteristics with histologic measures of breast tissue composition

Collagen fiber characteristics were not associated with participant characteristics among controls (Additional file 1: Table S4) and, therefore, models below were not adjusted for these factors.

Collagen fiber characteristics were associated with histologic measures of tissue composition extracted from H&E-stained biopsies (Table 1). Specifically, higher density of collagen fibers was significantly associated with greater proportions of stroma on the WSI in cases and controls (β = 0.57, 95% CI: 0.31, 0.84) and significantly inversely associated with proportions of fat on the WSI in controls (β = -0.49, 95% CI: -0.76, -0.21; Table 1). Among breast cancer cases, shorter, straighter, and thinner collagen fibers were associated with reduced amounts of epithelium on the WSI (*p* < 0.05) (Table 1). Among all women, fibers were significantly straighter with increasing amounts of fat on the WSI (β = 0.32, 95% CI: 0.02, 0.61) (Table 1).

**Table 1:** Associations of collagen fiber characteristics with histologic tissue composition metrics and radiologic mammographic density measures among women undergoing diagnostic breast biopsy

	All subjects (138 women, 243 ROI) β (95% CI)	*p *Value	Controls (73 women, 133 ROI) β (95% CI)	*p *Value	Cases (65 women, 110 ROI) β (95% CI)	*p *Value
*Tissue composition*						
Percent stroma on slide						
Fiber characteristic						
Average length (μm)	0.09 (− 0.14, 0.32)	0.462	0.15 (− 0.21, 0.50)	0.421	− 0.02 (− 0.29, 0.24)	0.869
Median straightness	− 0.14 (− 0.38, 0.10)	0.255	− 0.03 (− 0.43, 0.37)	0.875	− 0.24 (− 0.51, 0.03)	0.076
Average width (μm)	− 0.12 (− 0.37, 0.12)	0.323	− 0.15 (− 0.50, 0.20)	0.402	− 0.12 (− 0.41, 0.17)	0.403
Median alignment (in 44.91 μm × 44.91 μm)	0.07 (− 0.18, 0.32)	0.588	0.21 (− 0.18, 0.60)	0.297	− 0.12 (− 0.41, 0.16)	0.387
Density (number of fibers/100 μm^2^)	**0.57 (0.31, 0.84)**	**2.0 × 10**^**–5**^	**0.65 (0.33, 0.98)**	**7.9 × 10**^**–5**^	**0.45 (0.05, 0.85)**	**0.027**
Median orientation (°)	0.06 (− 0.25, 0.37)	0.717	− 0.14 (− 0.65, 0.38)	0.6	0.27 (− 0.07, 0.61)	0.124
Percent epithelium on slide						
Fiber characteristic						
Average length (μm)	− 0.17 (− 0.35, 0.02)	0.078	− 0.02 (− 0.23, 0.19)	0.867	**− 0.35 (− 0.64, − 0.07)**	**0.016**
Median straightness	**− 0.25 (− 0.44, − 0.06)**	0.012	− 0.06 (− 0.30, 0.18)	0.610	**− 0.41 (− 0.68, − 0.13)**	**3.6 × 10**^**–3**^
Average width (μm)	− 0.14 (− 0.32, 0.04)	0.129	0.02 (− 0.17, 0.21)	0.855	**− 0.4 (− 0.70, − 0.10)**	**9.6 × 10**^**–3**^
Median alignment (in 44.91 μm × 44.91 μm)	0.01 (− 0.15, 0.18)	0.885	0.06 (− 0.10, 0.22)	0.439	− 0.06 (− 0.38, 0.25)	0.692
Density (number of fibers/100 μm^2^)	− 0.12 (− 0.28, 0.04)	0.150	− 0.12 (− 0.30, 0.06)	0.193	− 0.14 (− 0.40, 0.13)	0.302
Median orientation (°)	0.17 (− 0.07, 0.4)	0.169	− 0.10 (− 0.32, 0.13)	0.392	**0.41 (0.06, 0.75)**	**0.021**
Percent fat on slide						
Fiber characteristic						
Average length (μm)	0.05 (− 0.23, 0.32)	0.745	− 0.11 (− 0.45, 0.23)	0.537	0.22 (− 0.21, 0.66)	0.309
Median straightness	**0.32 (0.02, 0.61)**	**0.034**	0.09 (− 0.30, 0.49)	0.645	**0.51 (0.10, 0.91)**	**0.014**
Average width (μm)	0.14 (− 0.15, 0.42)	0.352	0.06 (− 0.30, 0.41)	0.755	0.27 (− 0.19, 0.74)	0.249
Median alignment (in 44.91 μm × 44.91 μm)	− 0.03 (− 0.32, 0.25)	0.815	− 0.2 (− 0.59, 0.19)	0.318	0.18 (− 0.25, 0.60)	0.416
Density (number of fibers/100 μm^2^)	**− 0.49 (− 0.76, − 0.21)**	**6.6 × 10**^**–4**^	**− 0.49 (− 0.80, − 0.18)**	**2.1 × 10**^**–3**^	− 0.45 (− 0.92, 0.02)	0.059
Median orientation (°)	− 0.23 (− 0.56, 0.10)	0.167	0.09 (− 0.38, 0.55)	0.708	**− 0.53 (− 0.94, − 0.12)**	**0.011**
*MD measures*						
Global mammographic density						
% FGV						
Fiber characteristic						
Average length (μm)	− 0.04 (− 0.29, 0.21)	0.775	− 0.09 (− 0.49, 0.31)	0.654	6.9 × 10^–3^ (− 0.28, 0.30)	0.963
Median straightness	0.14 (− 0.12, 0.4)	0.283	0.04 (− 0.43, 0.51)	0.873	0.23 (− 0.05, 0.50)	0.102
Average width (μm)	− 0.11 (− 0.36, 0.14)	0.394	− 0.29 (− 0.64, 0.07)	0.114	0.16 (− 0.13, 0.45)	0.272
Median alignment (in 44.91 μm × 44.91 μm)	− 3.0 × 10^–3^ (− 0.24, 0.24)	0.980	0.07 (− 0.29, 0.43)	0.704	− 0.1 (− 0.39, 0.19)	0.517
Density (number of fibers/100 μm^2^)	0.23 (− 0.07, 0.52)	0.139	0.28 (− 0.14, 0.70)	0.193	0.15 (− 0.26, 0.56)	0.465
Median orientation (°)	− 0.06 (− 0.41, 0.3)	0.752	0.04 (− 0.49, 0.56)	0.894	− 0.1 (− 0.57, 0.36)	0.657
Dense volume						
Fiber characteristic						
Average length (μm)	0.12 (− 0.41, 0.65)	0.658	0.28 (− 0.48, 1.04)	0.466	− 0.07 (− 0.8, 0.67)	0.860
Median straightness	− 0.09 (− 0.61, 0.42)	0.723	0.39 (− 0.49, 1.27)	0.382	− 0.5 (− 1.07, 0.08)	0.092
Average width (μm)	0.12 (− 0.46, 0.7)	0.688	0.73 (− 5.5 × 10^–3^, 1.47)	0.052	**− 0.84 (− 1.54, − 0.15)**	**0.018**
Median alignment (in 44.91 μm × 44.91 μm)	− 0.02 (− 0.5, 0.46)	0.927	− 0.2 (− 0.92, 0.52)	0.578	0.2 (− 0.36, 0.77)	0.482
Density (number of fibers/100 μm^2^)	− 0.12 (− 0.63, 0.4)	0.664	0.2 (− 0.52, 0.93)	0.585	− 0.54 (− 1.21, 0.14)	0.119
Median orientation (°)	− 0.1 (− 0.88, 0.69)	0.810	0.87 (− 0.13, 1.87)	0.087	− 0.7 (− 1.63, 0.23)	0.138
Local mammographic density of the biopsy target						
% FGV						
Fiber characteristic						
Average length (μm)	− 0.02 (− 0.28, 0.23)	0.852	− 0.14 (− 0.54, 0.27)	0.506	0.07 (− 0.20, 0.35)	0.597
Median straightness	0.11 (− 0.16, 0.37)	0.433	− 0.07 (− 0.54, 0.41)	0.784	0.25 (− 0.03, 0.52)	0.077
Average width (μm)	− 0.17 (− 0.45, 0.11)	0.230	− 0.29 (− 0.69, 0.11)	0.155	0.01 (− 0.31, 0.34)	0.931
Median alignment (in 44.91 μm × 44.91 μm)	0.07 (− 0.18, 0.32)	0.571	0.12 (− 0.26, 0.49)	0.542	0.02 (− 0.29, 0.32)	0.921
Density (number of fibers/100 μm^2^)	**0.32 (0.02, 0.61)**	**0.035**	0.41 (− 0.01, 0.83)	0.056	0.20 (− 0.18, 0.58)	0.309
Median orientation (°)	− 0.09 (− 0.44, 0.26)	0.612	− 0.04 (− 0.60, 0.52)	0.886	− 0.10 (− 0.52, 0.32)	0.630
Dense volume						
Fiber characteristic						
Average length (μm)	− 0.07 (− 0.21, 0.08)	0.352	− 0.11 (− 0.31, 0.08)	0.258	− 0.02 (− 0.22, 0.19)	0.876
Median straightness	− 0.09 (− 0.23, 0.05)	0.220	− 0.04 (− 0.26, 0.18)	0.726	− 0.13 (− 0.32, 0.06)	0.180
Average width (μm)	− 0.08 (− 0.23, 0.08)	0.351	− 0.1 (− 0.32, 0.12)	0.366	− 0.03 (− 0.25, 0.19)	0.771
Median alignment (in 44.91 μm × 44.91 μm)	0.04 (− 0.11, 0.19)	0.625	0.09 (− 0.13, 0.32)	0.431	− 0.03 (− 0.21, 0.15)	0.747
Density (number of fibers/100 μm^2^)	− 0.04 (− 0.19, 0.12)	0.656	− 0.11 (− 0.34, 0.13)	0.384	0.06 (− 0.14, 0.26)	0.575
Median orientation (°)	0.11 (− 0.12, 0.33)	0.347	0.15 (− 0.17, 0.46)	0.355	0.06 (− 0.26, 0.37)	0.715
Local mammographic density in a peri− lesional 2 mm ring						
% FGV						
Fiber characteristic						
Average length (μm)	− 8.3 × 10^–3^ (− 0.28, 0.26)	0.952	− 0.14 (− 0.56, 0.28)	0.505	0.11 (− 0.19, 0.42)	0.471
Median straightness	0.17 (− 0.11, 0.44)	0.246	− 0.05 (− 0.54, 0.44)	0.851	**0.34 (0.05, 0.63)**	**0.022**
Average width (μm)	− 0.14 (− 0.43, 0.14)	0.328	− 0.31 (− 0.72, 0.10)	0.134	0.11 (− 0.23, 0.46)	0.511
Median alignment (in 44.91 μm × 44.91 μm)	0.06 (− 0.20, 0.33)	0.632	0.12 (− 0.27, 0.51)	0.555	− 4.1 × 10^–3^ (− 0.33, 0.32)	0.98
Density (number of fibers/100 μm^2^)	**0.35 (0.05, 0.64)**	**0.020**	0.42 (− 0.01, 0.85)	0.057	0.25 (− 0.11, 0.62)	0.17
Median orientation (°)	− 0.09 (− 0.46, 0.27)	0.614	− 0.04 (− 0.62, 0.53)	0.881	− 0.12 (− 0.57, 0.34)	0.613
Dense volume						
Fiber characteristic						
Average length (μm)	− 0.02 (− 0.08, 0.04)	0.529	− 0.04 (− 0.14, 0.06)	0.385	4.7 × 10^–3^ (− 0.06, 0.07)	0.884
Median straightness	− 0.01 (− 0.08, 0.05)	0.726	− 0.01 (− 0.12, 0.10)	0.850	− 0.01 (− 0.09, 0.07)	0.754
Average width (μm)	− 0.03 (− 0.10, 0.04)	0.438	− 0.03 (− 0.13, 0.07)	0.579	− 0.03 (− 0.11, 0.06)	0.541
Median alignment (in 44.91 μm × 44.91 μm)	0.02 (− 0.05, 0.08)	0.602	0.02 (− 0.07, 0.12)	0.607	7 × 10^–3^ (− 0.07, 0.09)	0.863
Density (number of fibers/100 μm^2^)	7.9 × 10^–3^ (− 0.07, 0.08)	0.832	− 3.1 × 10^–3^ (− 0.12, 0.11)	0.958	0.02 (− 0.06, 0.10)	0.603
Median orientation (°)	0.04 (− 0.05, 0.12)	0.384	0.07 (− 0.07, 0.22)	0.323	0.01 (− 0.09, 0.11)	0.804

Bold indicate statistically signficant associations

Based on generalized estimating equation (GEE) models with independent correlation structure

Outcomes of the models were the square root of either the tissue composition or the MD measure. Collagen fiber characteristics were the independent variables of the models. Models also included the diagnosis of the ROIs and the case–control status of the woman. β is per 1− standard deviation of the fiber characteristic

MD: Mammographic density; FGV: Fibroglandular volume

### Relation of collagen fiber characteristics with radiologic measures

As expected, the proportion of stroma on the WSI was significantly, positively associated with most of the global and local mammographic density measures (*p* < 0.05; Additional file 1: Table S5). Similarly, the proportion of fat on the WSI was significantly, inversely associated with most mammographic density measures (*p* < 0.05). Epithelial content on the WSI was positively associated with mammographic density, although this relationship did not reach statistical significance for most mammographic density measures (Additional file 1: Table S5).

Collagen fiber characteristics were largely unrelated to global mammographic density measures, with one exception: among breast cancer cases, fiber width decreased (i.e., became thinner) as absolute dense volume increased (β = − 0.84, 95% CI: − 1.54, − 0.15; Table 1). When examining relationships with localized mammographic density, we identified a positive association between collagen fiber density and percent FGV at both the biopsy target (β = 0.32, 95% CI: 0.02, 0.61) and in a 2 mm perilesional ring surrounding the biopsy target in the entire study population (β = 0.35, 95% CI: 0.05, 0.64; Table 1); similar, but non-significant, associations were observed in cases and controls separately. Among the breast cancer cases, straighter collagen fibers were also associated with higher percent FGV in a 2 mm ring surrounding the biopsy target (Table 1).

### Relation of collagen fiber characteristics with severity of ROI and biopsy diagnoses

Several collagen fiber characteristics significantly differed across the normal, benign and malignant ROIs in these diagnostic biopsies (Table 2). Specifically, compared with a normal ROI, longer collagen fibers were significantly associated with decreased odds of being in a benign (*p* = 4.18 × 10^–3^; Table 2) or a cancer (*p* = 1.85 × 10^–3^) ROI. Having straighter fibers was also significantly associated with decreased odds of being in a benign (*p* = 1.66 × 10^–3^) or in a cancer ROI (*p* = 0.015) compared with a normal ROI. Thicker fibers were significantly associated with increased odds of being in a cancer compared with a benign or normal ROI (*p* = 0.012 and *p* = 3.45 × 10^–3^, respectively). More aligned collagen fibers were significantly associated with increased odds of being in a cancer compared with a benign ROI (*p* = 3.50 × 10^–3^), and in a cancer compared with a normal ROI (*p* = 0.028). Higher collagen fiber density was also significantly associated with decreased odds of being in a benign compared with a normal ROI (*p* = 8.53 × 10^–5^), in a cancer compared with a benign ROI (*p* = 6.90 × 10^–3^), and in a cancer compared with a normal ROI (*p* = 7.58 × 10^–3^). While collagen fiber characteristics were associated with severity of diagnosis at the ROI level, they were not associated with overall diagnosis of the woman (Table 3).

**Table 2:** Associations of collagen fiber characteristics with the diagnoses of the regions of interest (ROIs) on diagnostic H&E-stained biopsies among women undergoing diagnostic breast biopsy

	Benign ROI vs. normal ROI (ref)		Cancer ROI vs. benign ROI (ref)		Cancer ROI vs. normal ROI (ref)	
	OR (95% CI)	*p *Value	OR (95% CI)	*p *Value	OR (95% CI)	*p *Value
Average length (μm)	**0.67 (0.51, 0.88)**	**4.18 × 10**^**–3**^	0.78 (0.56, 1.10)	0.153	**0.43 (0.25, 0.73)**	**1.85 × 10**^**–3**^
Median straightness	**0.62 (0.46, 0.83)**	**1.66 × 10**^**–3**^	0.83 (0.60, 1.16)	0.275	**0.59 (0.39, 0.90)**	**0.015**
Average width (μm)	0.83 (0.65, 1.07)	0.151	**0.56 (0.36, 0.88)**	**0.012**	**0.48 (0.31, 0.75)**	**1.45 × 10**^**–3**^
Median alignment (in 44.91 μm × 44.91 μm)	0.9 (0.68, 1.20)	0.484	**2.13 (1.28, 3.54)**	**3.50 × 10**^**–3**^	**1.88 (1.07, 3.29)**	**0.028**
Density (number of fibers/100 μm^2^)	**0.52 (0.38, 0.72)**	**8.53 × 10**^**–5**^	**0.54 (0.34, 0.84)**	**6.90 × 10**^**–3**^	**0.20 (0.06, 0.65)**	**7.58 × 10**^**–3**^
Median orientation (°)	0.79 (0.48, 1.3)	0.353	0.94 (0.6, 1.47)	0.782	1.17 (0.68, 2.03)	0.569

Bold indicate statistically signficant associations

Based on generalized estimating equation (GEE) models with independent correlation structure. Outcomes of the models were the diagnosis of the ROI. Collagen fiber characteristics were the independent variables of the models. Models also included the case–control status of the woman. OR is per 1-standard deviation of the fiber characteristic

Ref: reference category. OR: Odds ratio

**Table 3:** Associations of collagen fiber characteristics with the overall biopsy diagnosis among women undergoing diagnostic breast biopsy

	Fiber characteristics measured in all ROIs		Fiber characteristics measured in benign and cancer ROIs only	
	OR (95% CI)	*p *Value	OR (95% CI)	*p *Value
Average length (μm)	1.17 (0.80, 1.70)	0.416	1.01 (0.61, 1.68)	0.963
Median straightness	0.97 (0.65, 1.44)	0.881	0.96 (0.61, 1.51)	0.845
Average width (μm)	1.08 (0.76, 1.53)	0.678	1.03 (0.70, 1.52)	0.878
Median alignment (in 44.91 μm × 44.91 μm)	0.93 (0.66, 1.31)	0.675	0.91 (0.60, 1.40)	0.68
Density (number of fibers/100 μm^2^)	1.24 (0.86, 1.78)	0.248	1.12 (0.73, 1.72)	0.618
Median orientation (°)	1.43 (0.83, 2.47)	0.192	1.78 (0.93, 3.40)	0.08

Based on generalized estimating equation (GEE) models with independent correlation structure. Outcomes of the models were the overall diagnosis of the woman. Collagen fiber characteristics were the independent variables of the models. Models also included the diagnosis of the ROIs. OR is per 1-standard deviation of the fiber characteristic

We also evaluated whether collagen fiber characteristics in cancerous ROIs were associated with breast tumor characteristics (Additional file 1: Table S6). Straighter collagen fibers were significantly associated with grade III compared with grade I and II cancers (*p* = 0.04). Higher density collagen fibers were significantly associated with larger tumors (≥ 1 cm *vs* < 1 cm) (*p* = 0.04).

### Relation of the tumor associated collagen signature (TACS)-3 with severity of ROI diagnosis

Finally, each ROI was scored for the presence or absence of TACS-3 by three reviewers. Agreement by all three was reached for 230 out of 243 ROIs (94.6%) and by two reviewers in the remaining 13 ROIs. TACS-3 was present in only 20 (8.2%) of ROIs, which as expected were predominantly malignant: fifteen had a cancer diagnosis (75%), two had a benign diagnosis in cancer-free patients (10%), and three ROIs had a normal diagnosis also in cancer-free patients (15%). Due to the low proportion of TACS-3 in this population, we did not evaluate the relationship of this signature with measures of breast tissue composition, mammographic density, or other characteristics. We did not observe any association between TACS-3 and tumor characteristics.

## Discussion

In this population of women undergoing diagnostic image-guided biopsy, collagen fiber density was significantly and positively associated with local, but not global, volumetric percent mammographic density. Using high-resolution SHG microscopy of diagnostic breast biopsies, we found that other collagen fiber characteristics were not significantly associated with mammographic density. However, collagen fiber features, including length, straightness, width, alignment, and density, were significantly associated with lesion severity. As lesion severity increased from normal to benign to malignant, fibrillar collagen density decreased and fibers tended to be shorter, straighter, thinner, and more aligned with one another. Although stromal collagen microarchitecture may not translate into macroscopic measures of mammographic density, collagen features may be a marker of cancer risk among women referred for biopsy based on abnormal breast imaging.

The lack of statistically significant associations between collagen fiber architecture, other than collagen fiber density, and global measures of mammographic density, suggests that the coarse resolution of global mammographic density measures may not capture the microscopic resolution of collagen organization on a diagnostic breast biopsy. A previous study found increased collagen deposition and organization in breast tissues sections taken from regions of higher mammographic density tissue slices, which were resected from 41 prophylactic mastectomies, and then X-rayed to determine their radiological appearance [8]. This approach for measuring mammographic density may have been closer to the scale of collagen architecture, potentially explaining their positive findings. However, it is unclear how these findings would translate to in vivo density measures of the entire breast. Another study, using tissues from prophylactic mastectomies in premenopausal patients, also found higher collagen density and thicker collagen fibers in patients with higher mammographic density (*N* = 12) compared with patients with low mammographic density (*N* = 10) based on the Breast Imaging-Reporting and Data System (BI-RADS) density assessment taken before the prophylactic surgery [10]. We observed greater fiber width associated with global and local percent FGV in cancer cases, but not in controls, although findings did not reach statistical significance. A study of postmenopausal women undergoing research biopsies targeted at areas of high and low mammographic density found that collagen fibers were more aligned and thicker in six patients with high compared to six patients with low mammographic density [9]; however, unexpectedly, that study did not find stromal content to be correlated with mammographic density. Prior work in this and other study populations has shown strong positive associations between stromal content and global density measures [6–8, 20, 27]. We also observed a significant positive association between % FGV and proportion of the stroma on the slide in our analytic population and found that stromal proportion was positively associated collagen fiber density, lending internal validity to our results.

Although the association between collagen fiber density and measures of global volumetric mammographic density was not statistically significant in our study, the association of collagen fiber density with percent FGV was in the same positive direction as previously reported for smaller studies, which used a variety of methods to measure collagen and its relationships with percent area density [7, 10] or visually assessed categories of dense area [6, 8]. The SHG imaging technology we employed in this study images all fibrillar collagens, including Type I collagen, the most common subtype in the breast [28]. Some prior studies measured collagen using Masson’s trichrome stained tissue sections [7, 8]; while Masson’s trichrome stain is useful to assess abundance of amorphous collagen [9], it is not specific to fibrillar collagen. A smaller study that measured collagen in research biopsies using both Masson’s trichrome stain and Picrosirius red (PSR) staining, which is specific for fibrillar collagen [29], found that volumetric mammographic density was only correlated with PSR collagen and not Masson’s trichrome-stained collagen [9]. Future studies should further examine which is the most robust and reliable measure of collagen content. Ideally, such a measure should be high-throughput for applicability in large-scale epidemiological studies.

We identified a novel association between collagen fiber density and localized mammographic density measures at and surrounding the biopsy target. This finding is important because it may provide information about tissue remodeling at the location of premalignant and malignant breast abnormalities. Prior studies examining associations between collagen and breast density have primarily focused on radiological determinations from X-rays of breast tissue sections [8] or global area measures [7, 10]. However, it may be difficult to see local, fiber-level features reflected in global mammographic density measures averaged across the entire breast. Even though mammographic density is thought to be a general marker of risk, it is clear that there is heterogeneity in the distribution of density and that parenchymal patterns may be important to understand. More localized density measures and radiologic features (like texture features) may more accurately reflect characteristics of the ECM and provide different information about breast cancer risk than a global average density measure.

It is important to understand how collagen fiber organization contributes to increased risk of breast precursor lesions and breast cancer. Our design allowed us to study the relationship between collagen architecture and lesion severity at the ROI level in the biopsy WSIs. It is possible that localized collagen content may increase risk via prolonged inflammatory cytokine or mechano-sensitive signaling, leading to higher risk of developing cancer [30, 31]. We found that multiple collagen fiber characteristics were related to lesion severity. In particular, we found that decreasing collagen fiber length, straightness, width, and fiber density and increased fiber alignment were significantly associated with increased lesion severity of the ROI. Interestingly, decreasing fiber length and straightness were found to be associated with the transition from normal to BBD, whereas decreasing fiber width and increasing fiber alignment were associated with the transition from BBD to cancer. Thus, the structural and organizational properties of collagen fibers seem to change with the onset of benign breast disease, and perhaps the changes in collagen length and straightness set the stage for changes in fiber width and alignment. Importantly, previous work has shown that several collagen features, such as decreased width, density, and straightness, were associated with increased risk of recurrence after ductal carcinoma in situ [13], suggesting that remodeling of tissue near a precursor lesion is also important for breast cancer outcomes. We also observed that collagen fiber density and straightness around a cancer lesion were associated with greater tumor size and grade, respectively. While collagen micro-organization was related to the diagnosis of the ROI, it was not associated with the overall diagnosis of the woman. This may be due to the fact that collagen organization is a local process that occurs during the transformation of the lesion from normal to cancer and multiple processes may occur simultaneously [10].

The collagen signature TACS-3 was previously defined in tissues surrounding breast cancer tumors and characterized as bundles of collagen fibers straightened and aligned that were perpendicular to the tumor boundary[14, 15]. To date, TACS-3 has only been evaluated in cancers, and it was unclear whether TACS-3 might also provide information regarding early stages of carcinogenesis. In our study, we found that the TACS-3 collagen signature was absent in the majority of normal and benign samples. For the regions surrounding cancer cases, TACS-3 was present in only 15 regions out of 56, and 11 of these 15 tumors were ≥ 1 cm, as previously observed [14]. Our results suggest that TACS-3 may be a later event in cancer progression and invasion.

Our study is one of the largest and most comprehensive to date to evaluate breast cancer risk factor relationships with quantitative collagen fiber features. We used SHG imaging technology to quantify multiple collagen fiber characteristics on H&E slides without additional tissue processing; however, this method is labor-intensive precluding a larger-scale study design. Strengths of our study include the detailed data on participant characteristics, collagen fiber features, a range of biopsy diagnoses and reliable volumetric measures of global and localized mammographic density, at and surrounding the biopsy target. Finally, we were able to evaluate collagen microarchitecture in relation to biopsy lesion severity.

Collagen is a major component of the stromal tissue surrounding breast ducts, where most breast cancers arise. Laboratory studies have shown that fibrillar collagen plays a key role in promoting tumor initiation and metastasis [12]. We found that fibrillar collagen density is associated with local mammographic density among women referred for biopsy based on abnormal breast imaging, which may indicate local tissue reorganization in the setting of BBD and breast cancer. In addition, several collagen fiber features were related to lesion severity, suggesting opportunities for future research integrating collagen microarchitecture with other features of the microenvironment observed in diagnostic biopsy sections as biomarkers of breast cancer risk. However, there is a need to determine robust, reliable and high-throughput methods to measure fibrillar collagen that can be used in large-scale epidemiological studies and clinical settings.

## Summary and conclusions

Elevated mammographic breast density is a strong breast cancer risk factor with poorly understood etiology. Increased deposition of collagen, one of the main fibrous proteins present in breast stroma, has been associated with increased mammographic density. Using novel second harmonic generation imaging to quantify individual collagen fiber features within in routinely prepared, H&E-stained slides from diagnostic biopsies, we examined their relationships with local and mammographic density volumes and lesion severity. We found that collagen fiber density was positively associated with local, but not global, mammographic density. Importantly, we found multiple collagen fiber features to be significantly associated with the breast biopsy diagnosis. Specifically, as the severity of the breast biopsy diagnosis increased, collagen fibers tended to be less dense, shorter, straighter, thinner, and more aligned with one another. Our findings suggest that collagen fiber features may be markers of cancer risk and/or progression among women undergoing image-guided breast biopsy.

## Supplementary Information

**Additional file 1.** Description of the characteristics of the women included in the study, the tumor characteristics for the cases, and the ROIs. It also includes tables with associations between participant characteristics and collagen fiber characteristics, mammographic density measures and histolic tissue composition, and between collagen fiber characteristics and tumor characteristics.

## Data Availability

The datasets generated or analyzed during the current study are not publicly available due to data privacy of patients. The authors will make the data available upon reasonable request.

## References

[1] Boyd NF, Martin LJ, Yaffe MJ, Minkin S (2011). Mammographic density and breast cancer risk: current understanding and future prospects. Breast Cancer Res BCR.

[2] McCormack VA, dos Santos SI (2006). Breast density and parenchymal patterns as markers of breast cancer risk: a meta-analysis. Cancer Epidemiol Biomarkers Prev.

[3] Martin LJ, Boyd NF: Mammographic density. Potential mechanisms of breast cancer risk associated with mammographic density: hypotheses based on epidemiological evidence. *Breast cancer research : BCR* 2008, 10(1):201. 10.1186/bcr1831 PMC2374950 18226174

[4] Abubakar M, Fan S, Bowles EA, Widemann L, Duggan MA, Pfeiffer RM, Falk RT, Lawrence S, Richert-Boe K, Glass AG *et al*: Relation of Quantitative Histologic and Radiologic Breast Tissue Composition Metrics with Invasive Breast Cancer Risk. *JNCI Cancer Spectr (In press)* 2021:2020.2011.2012.20230623. 10.1093/jncics/pkab015 PMC8103888 33981950

[5] Frantz C, Stewart KM, Weaver VM (2010). The extracellular matrix at a glance. J Cell Sci.

[6] Alowami S, Troup S, Al-Haddad S, Kirkpatrick I, Watson PH (2003). Mammographic density is related to stroma and stromal proteoglycan expression. Breast Cancer Res.

[7] Li T, Sun L, Miller N, Nicklee T, Woo J, Hulse-Smith L, Tsao MS, Khokha R, Martin L, Boyd N (2005). The association of measured breast tissue characteristics with mammographic density and other risk factors for breast cancer. Cancer Epidemiol Biomarkers Prev.

[8] Huo CW, Chew G, Hill P, Huang D, Ingman W, Hodson L, Brown KA, Magenau A, Allam AH, McGhee E (2015). High mammographic density is associated with an increase in stromal collagen and immune cells within the mammary epithelium. Breast Cancer Res.

[9] McConnell JC, O’Connell OV, Brennan K, Weiping L, Howe M, Joseph L, Knight D, O’Cualain R, Lim Y, Leek A (2016). Increased peri-ductal collagen micro-organization may contribute to raised mammographic density. Breast Cancer Res.

[10] Northey JJ, Barrett AS, Acerbi I, Hayward MK, Talamantes S, Dean IS, Mouw JK, Ponik SM, Lakins JN, Huang PJ (2020). Stiff stroma increases breast cancer risk by inducing the oncogene ZNF217. J Clin Invest.

[11] Huang X, Reye G, Momot KI, Blick T, Lloyd T, Tilley WD, Hickey TE, Snell CE, Okolicsanyi RK, Haupt LM (2020). Heparanase promotes syndecan-1 expression to mediate fibrillar collagen and mammographic density in human breast tissue cultured ex vivo. Front Cell Dev Biol.

[12] Provenzano PP, Inman DR, Eliceiri KW, Knittel JG, Yan L, Rueden CT, White JG, Keely PJ (2008). Collagen density promotes mammary tumor initiation and progression. BMC Med.

[13] Sprague BL, Vacek PM, Mulrow SE, Evans MF, Trentham-Dietz A, Herschorn SD, James TA, Surachaicharn N, Keikhosravi A, Eliceiri KW (2021). Collagen organization in relation to ductal carcinoma in situ pathology and outcomes. Cancer Epidemiol Biomarkers Prev.

[14] Conklin MW, Eickhoff JC, Riching KM, Pehlke CA, Eliceiri KW, Provenzano PP, Friedl A, Keely PJ (2011). Aligned collagen is a prognostic signature for survival in human breast carcinoma. Am J Pathol.

[15] Provenzano PP, Eliceiri KW, Campbell JM, Inman DR, White JG, Keely PJ (2006). Collagen reorganization at the tumor-stromal interface facilitates local invasion. BMC Med.

[16] Gierach GL, Geller BM, Shepherd JA, Patel DA, Vacek PM, Weaver DL, Chicoine RE, Pfeiffer RM, Fan B, Mahmoudzadeh AP (2014). Comparison of mammographic density assessed as volumes and areas among women undergoing diagnostic image-guided breast biopsy. Cancer Epidemiol Biomarkers Prev.

[17] Malkov S, Wang J, Kerlikowske K, Cummings SR, Shepherd JA (2009). Single x-ray absorptiometry method for the quantitative mammographic measure of fibroglandular tissue volume. Med Phys.

[18] Gierach GL, Patel DA, Pfeiffer RM, Figueroa JD, Linville L, Papathomas D, Johnson JM, Chicoine RE, Herschorn SD, Shepherd JA (2016). Relationship of terminal duct lobular unit involution of the breast with area and volume mammographic densities. Cancer Prev Res (Phila).

[19] Ehteshami Bejnordi B, Mullooly M, Pfeiffer RM, Fan S, Vacek PM, Weaver DL, Herschorn S, Brinton LA, van Ginneken B, Karssemeijer N (2018). Using deep convolutional neural networks to identify and classify tumor-associated stroma in diagnostic breast biopsies. Mod Pathol.

[20] Mullooly M, Ehteshami Bejnordi B, Pfeiffer RM, Fan S, Palakal M, Hada M, Vacek PM, Weaver DL, Shepherd JA, Fan B (2019). Application of convolutional neural networks to breast biopsies to delineate tissue correlates of mammographic breast density. NPJ Breast Cancer.

[21] Bredfeldt JS, Liu Y, Conklin MW, Keely PJ, Mackie TR, Eliceiri KW (2014). Automated quantification of aligned collagen for human breast carcinoma prognosis. J Pathol Inform.

[22] Linkert M, Rueden CT, Allan C, Burel JM, Moore W, Patterson A, Loranger B, Moore J, Neves C, Macdonald D (2010). Metadata matters: access to image data in the real world. J Cell Biol.

[23] Preibisch S, Saalfeld S, Tomancak P (2009). Globally optimal stitching of tiled 3D microscopic image acquisitions. Bioinformatics.

[24] Liu Y, Keikhosravi A, Pehlke CA, Bredfeldt JS, Dutson M, Liu H, Mehta GS, Claus R, Patel AJ, Conklin MW (2020). Fibrillar Collagen Quantification With Curvelet Transform Based Computational Methods. Front Bioeng Biotechnol.

[25] Liu Y, Eliceiri KW: Quantifying fibrillar collagen organization with curvelet transform-based tools. J Vis Exp 2020(165). 10.3791/61931 33252107

[26] Bredfeldt JS, Liu Y, Pehlke CA, Conklin MW, Szulczewski JM, Inman DR, Keely PJ, Nowak RD, Mackie TR, Eliceiri KW (2014). Computational segmentation of collagen fibers from second-harmonic generation images of breast cancer. J Biomed Opt.

[27] Sun X, Gierach GL, Sandhu R, Williams T, Midkiff BR, Lissowska J, Wesolowska E, Boyd NF, Johnson NB, Figueroa JD (2013). Relationship of mammographic density and gene expression: analysis of normal breast tissue surrounding breast cancer. Clin Cancer Res.

[28] Chen X, Nadiarynkh O, Plotnikov S, Campagnola PJ (2012). Second harmonic generation microscopy for quantitative analysis of collagen fibrillar structure. Nat Protoc.

[29] Junqueira LC, Bignolas G, Brentani RR (1979). Picrosirius staining plus polarization microscopy, a specific method for collagen detection in tissue sections. Histochem J.

[30] Esbona K, Inman D, Saha S, Jeffery J, Schedin P, Wilke L, Keely P (2016). COX-2 modulates mammary tumor progression in response to collagen density. Breast Cancer Res BCR.

[31] Provenzano PP, Inman DR, Eliceiri KW, Keely PJ (2009). Matrix density-induced mechanoregulation of breast cell phenotype, signaling and gene expression through a FAK–ERK linkage. Oncogene.

