# Supplementary material for: Mammary collagen architecture and its association with mammographic density and lesion severity among women undergoing image-guided breast biopsy
Source: Breast Cancer Res. 2021 Nov 9;23:105. doi: 10.1186/s13058-021-01482-z (PMC8579610; doi:10.1186/s13058-021-01482-z)
Supplement: Supplementary file 1 — Additional file 1. Description of the characteristics of the women included in the study, the tumor characteristics for the cases, and the ROIs. It also includes tables with associations between participant characteristics and collagen fiber characteristics, mammographic density measures and histolic tissue composition, and between collagen fiber characteristics and tumor characteristics. [file 13058_2021_1482_MOESM1_ESM.docx]

**Table S1.** Characteristics of women undergoing diagnostic breast biopsy by case-control status^*^.

| **Characteristics** | **Overall**  **(N=138)** | **Controls**  **(N=73)** | **Cases**  **(N=65)** | **P-value^§^** |
| --- | --- | --- | --- | --- |
| *Age (years), n (%)* |  |  |  | 0.89 |
| 39-44 | 15 (10.9) | 8 (11.0) | 7 (10.8) |  |
| 45-49 | 30 (21.7) | 18 (24.7) | 12 (18.5) |  |
| 50-54 | 40 (29.0) | 21 (28.8) | 19 (29.2) |  |
| 55-59 | 35 (25.4) | 18 (24.7) | 17 (26.2) |  |
| 60-65 | 18 (13.0) | 8 (11.0) | 10 (15.4) |  |
|  |  |  |  |  |
| *Body mass index (BMI) (Kg/m^2^), n (%)* |  |  |  | 0.97 |
| <25 | 64 (46.4) | 33 (45.2) | 31 (47.7) |  |
| 25-<30 | 25 (18.1) | 14 (19.2) | 11 (16.9) |  |
| ≥30 | 49 (35.5) | 26 (35.6) | 23 (35.4) |  |
|  |  |  |  |  |
| *Menopausal status, n (%)* |  |  |  | 0.87 |
| Premenopausal | 65 (47.1) | 35 (47.9) | 30 (46.2) |  |
| Postmenopausal | 73 (52.9) | 38 (52.1) | 35 (53.8) |  |
|  |  |  |  |  |
| *Age at menarche (years), n (%)* |  |  |  | 0.82 |
| ≤12 | 49 (36.0) | 25 (35.2) | 24 (36.9) |  |
| 13 | 52 (38.2) | 26 (36.6) | 26 (40.0) |  |
| ≥14 | 35 (25.7) | 20 (28.2) | 15 (23.1) |  |
|  |  |  |  |  |
| *Parity, n (%)* |  |  |  | 0.51 |
| Nulliparous | 32 (23.2) | 18 (24.7) | 14 (21.5) |  |
| 1 | 21 (15.2) | 8 (11.0) | 13 (20.0) |  |
| 2 | 56 (40.6) | 30 (41.1) | 26 (40.0) |  |
| ≥3 | 29 (21.0) | 17 (23.3) | 12 (18.5) |  |
|  |  |  |  |  |
| *Age at first birth (years), n (%)* |  |  |  | **0.01** |
| Nulliparous | 32 (23.5) | 18 (25.0) | 14 (21.9) |  |
| <30 | 76 (55.9) | 46 (63.9) | 30 (46.9) |  |
| ≥30 | 28 (20.6) | 8 (11.1) | 20 (31.2) |  |
|  |  |  |  |  |
| *Smoking (100+ cigarettes/lifetime), n (%)* |  |  |  | 0.31 |
| Never | 67 (48.9) | 39 (53.4) | 28 (43.8) |  |
| Ever | 70 (51.1) | 34 (46.6) | 36 (56.2) |  |
|  |  |  |  |  |
| *Family history of breast cancer, n (%)* |  |  |  | 1.00 |
| No | 99 (71.7) | 52 (71.2) | 47 (72.3) |  |
| Yes | 39 (28.3) | 21 (28.8) | 18 (27.7) |  |
|  |  |  |  |  |
| *Breast biopsy prior to enrollment, n (%)* |  |  |  | 0.38 |
| No | 88 (63.8) | 44 (60.3) | 44 (67.7) |  |
| Yes | 50 (36.2) | 29 (39.7) | 21 (32.3) |  |
|  |  |  |  |  |
| *Biopsy type, n (%)* |  |  |  | 0.09 |
| Ultrasound-guided | 59 (43.1) | 26 (36.1) | 33 (50.8) |  |
| Stereotactic-guided | 78 (56.9) | 46 (63.9) | 32 (49.2) |  |
|  |  |  |  |  |
| *Worst pathologic diagnosis, n (%)* |  |  |  | - |
| Benign | 30 (21.7) | 30 (41.1) | - |  |
| Proliferative | 35 (25.4) | 35 (47.9) | - |  |
| Proliferative with atypia | 8 ( 5.8) | 8 (11.0) | - |  |
| *In situ* (LCIS or DCIS) | 32 (23.2) | - | 32 (49.2) |  |
| Invasive | 33 (23.9) | - | 33 (50.8) |  |
|  |  |  |  |  |
| *Global measures of mammographic density in affected breast, mean (SD)* |  |  |  |  |
| % FGV | 36.7 (21.5) | 36.7 (22.7) | 34 (18) | 0.44 |
| Dense volume | 201.6 (99.5) | 201.0 (106.3) | 202.2 (92.0) | 0.94 |
|  |  |  |  |  |
| *Local measures of mammographic density at biopsy site, mean (SD)* |  |  |  |  |
| % FGV | 44.7 (24.5) | 44.3 (26.6) | 45.2 (22.2) | 0.83 |
| Dense volume | 4.2 (5.4) | 3.8 (6.1) | 4.6 (4.3) | 0.35 |
| Volume | 10.1 (11.4) | 8.2 (10.2) | 12.2 (12.3) | **0.04** |
| Radius lesion (cm) | 0.7 (0.4) | 0.6 (0.4) | 0.8 (0.4) | **0.03** |
|  |  |  |  |  |
| *Local measures of mammographic density in a 2mm ring surrounding biopsy site, mean (SD)* |  |  |  |  |
| % FGV | 41.9 (24.7) | 41.6 (26.7) | 42.1 (22.4) | 0.91 |
| Dense volume | 1.9 (1.3) | 1.8 (1.4) | 2.0 (1.1) | 0.23 |
| Volume | 5.0 (2.8) | 4.6 (2.5) | 5.6 (2.9) | **0.04** |

FGV: Fibroglandular volume.

^*^Cases were women who received a biopsy diagnosis of either *in situ* (n=32) or invasive (n=33) cancer. Controls were women diagnosed with benign breast disease.

^§^Based on the Fisher’s exact test comparing non-continuous characteristics for cases and controls. Based on a t-test for continuous characteristics.

**Table S2.** Tumor characteristics of breast cancer cases diagnosed on a clinically-indicated breast biopsy.

| **Characteristics** | **Cases**  **(N=65)**  **n (%)** |
| --- | --- |
| *Grade* |  |
| DCIS |  |
| I | 3 (10.3) |
| II | 12 (41.4) |
| III | 14 (48.3) |
| Invasive cancer |  |
| I | 7 (21.3) |
| II | 19 (63.3) |
| III | 4 (13.3) |
|  |  |
| *Lesion size (at surgery)* |  |
| <1 cm | 11 (30.6) |
| ≥1 cm | 25 (69.4) |
|  |  |
| *Histologic type* |  |
| Ductal | 54 (87.1) |
| Lobular | 6 ( 9.7) |
| Other | 2 ( 6.7) |
|  |  |
| *ER status* |  |
| Negative | 13 (22.8) |
| Positive | 44 (77.2) |
|  |  |
| *PR status* |  |
| Negative | 15 (26.3) |
| Positive | 42 (73.7) |

**Table S3.** Descriptive statistics of number regions of interest (ROIs).

| **Characteristic** | **Controls** | **Cases** | **P-value^§^** |
| --- | --- | --- | --- |
| *All ROIs* |  |  |  |
| Number of subjects (N=138), n | 73 | 65 |  |
|  |  |  |  |
| Number of ROI (N=243), n | 133 | 110 |  |
|  |  |  |  |
| Number of ROI on slide per subject, n (%) |  |  | 0.15 |
| 1 | 16 (21.9) | 20 (30.8) |  |
| 2 | 54 (74.0) | 45 (69.2) |  |
| 3 | 3 ( 4.1) | - |  |
|  |  |  |  |
| Diagnosis of ROI on slide, n (%) |  |  | <2.2×10^-16^ |
| Normal | 62 (46.6) | 15 (13.6) |  |
| Benign | 71 (53.4) | 39 (35.5) |  |
| Cancer | - | 56 (50.9) |  |
|  |  |  |  |
| Number of ROI on slides and diagnosis of the spot, n (%) |  |  |  |
| 1 |  |  | 3.7×10^-5^ |
| Normal | 7 (43.8) | 1 ( 5.0) |  |
| Benign | 9 (56.2) | 6 (30.0) |  |
| Cancer | - | 13 (65.0) |  |
| 2 |  |  | <2.2×10^-16^ |
| Normal, normal | 1 ( 1.9) | - |  |
| Normal, benign | 47 (87.0) | - |  |
| Normal, cancer | - | 14 (31.1) |  |
| Benign, benign | 6 (11.1) | 2 ( 4.4) |  |
| Benign, cancer | - | 29 (64.4) |  |
| 3 |  |  | - |
| Normal, normal, normal | 1 (33.3) | - |  |
| Normal, normal, benign | 1 (33.3) | - |  |
| Normal, benign, benign | 1 (33.3) | - |  |
| *Subset of ROIs used for orientation measures of collagen^*^* |  |  |  |
| Number of subjects (N=82), n | 44 | 38 |  |
|  |  |  |  |
| Number of ROI (N=127), n | 72 | 55 |  |
|  |  |  |  |
| Number of ROI on slide per subject, n (%) |  |  | 0.30 |
| 1 | 16 (36.4) | 18 (47.4) |  |
| 2 | 25 (56.8) | 20 (52.6) |  |
| 3 | 3 ( 6.8) | - |  |
|  |  |  |  |
| Diagnosis of ROI on slide, n (%) |  |  | 1.3×10^-12^ |
| Normal | 36 (50.0) | 9 (16.4) |  |
| Benign | 36 (50.0) | 19 (34.5) |  |
| Cancer | - | 27 (49.1) |  |
|  |  |  |  |
| Number of ROI on slides and diagnosis of the spot, n (%) |  |  |  |
| 1 |  |  | 3.5×10^-5^ |
| Normal | 7 (43.8) | 1 ( 5.6) |  |
| Benign | 9 (56.2) | 5 (27.8) |  |
| Cancer | - | 12 (66.7) |  |
| 2 |  |  | 6.3×10^-13^ |
| Normal, normal | 1 ( 4.0) | - |  |
| Normal, benign | 22 (88.0) | - |  |
| Normal, cancer | - | 8 (40.0) |  |
| Benign, benign | 2 ( 8.0) | 2 (10.0) |  |
| Benign, cancer | - | 10 (50.0) |  |
| 3 |  |  | - |
| Normal, normal, normal | 1 (33.3) | - |  |
| Normal, normal, benign | 1 (33.3) | - |  |
| Normal, benign, benign | 1 (33.3) | - |  |

^§^Based on a Fisher’s exact test or the Wilcoxon rank sum test to compare distributions of number of squares/fibers.

^*^See methods for stitched data.

**Table S4.** Associations between participant characteristics and collagen fiber characteristics among controls^*^.

|  | β (95% CI) ^§^ |
| --- | --- |
| *Average length (μm)* |  |
| Age (cont.) | -4.4×10^-3^ (-0.02, 0.02) |
| BMI (cont.) | -8.1e×10^-5^ (-0.02, 0.02) |
| BMI (cat., trend) | -0.03 (-0.19, 0.14) |
| Menopausal status (no/yes) | 0.06 (-0.21, 0.32) |
| Age at menarche (cat., trend) | -0.05 (-0.25, 0.15) |
| Parity (no/yes) | 0.08 (-0.2, 0.36) |
| Age at first birth (cat., trend) | 0.04 (-0.14, 0.22) |
| Smoking (never/ever) | 0.09 (-0.17, 0.34) |
| Family history (no/yes) | -0.06 (-0.36, 0.24) |
| Prior breast biopsy (no/yes) | -0.07 (-0.33, 0.19) |
| *Median straightness* |  |
| Age (cont.) | 4.1×10^-3^ (-0.01, 0.02) |
| BMI (cont.) | -4.5×10^-3^ (-0.02, 0.01) |
| BMI (cat., trend) | -0.03 (-0.16, 0.1) |
| Menopausal status (no/yes) | 0.2 (-0.01, 0.41) |
| Age at menarche (cat., trend) | -0.12 (-0.28, 0.03) |
| Parity (no/yes) | 0.02 (-0.24, 0.29) |
| Age at first birth (cat., trend) | 0.05 (-0.12, 0.21) |
| Smoking (never/ever) | 9.5×10^-3^ (-0.21, 0.23) |
| Family history (no/yes) | -0.01 (-0.26, 0.24) |
| Prior breast biopsy (no/yes) | 0.1 (-0.12, 0.33) |
| *Average width (μm)* |  |
| Age (cont.) | -3.6×10^-4^ (-2.6×10^-3^, 1.9×10^-3^) |
| BMI (cont.) | 9.9×10^-4^ (-1.3×10^-3^, 3.3×10^-3^) |
| BMI (cat., trend) | 6.7×10^-3^ (-0.01, 0.02) |
| Menopausal status (no/yes) | -8.3×10^-4^ (-0.03, 0.03) |
| Age at menarche (cat., trend) | -4.2×10^-3^ (-0.02, 0.01) |
| Parity (no/yes) | -0.01 (-0.05, 0.02) |
| Age at first birth (cat., trend) | -1.9×10^-3^ (-0.03, 0.02) |
| Smoking (never/ever) | 9.5×10^-3^ (-0.02, 0.04) |
| Family history (no/yes) | -3.7×10^-3^ (-0.04, 0.03) |
| Prior breast biopsy (no/yes) | -1.9×10^-3^ (-0.03, 0.03) |
| *Median alignment (in 44.91μm×44.91μm)* |  |
| Age (cont.) | -0.11 (-0.39, 0.17) |
| BMI (cont.) | -0.17 (-0.47, 0.12) |
| BMI (cat., trend) | -1.14 (-3.43, 1.14) |
| Menopausal status (no/yes) | 0.11 (-3.66, 3.89) |
| Age at menarche (cat., trend) | 0.16 (-2.2, 2.52) |
| Parity (no/yes) | **4.47 (0.49, 8.45)** |
| Age at first birth (cat., trend) | 2.62 (-0.06, 5.3) |
| Smoking (never/ever) | 0.4 (-3.26, 4.07) |
| Family history (no/yes) | 0.31 (-3.35, 3.97) |
| Prior breast biopsy (no/yes) | -2.32 (-5.96, 1.32) |
| *Density (number of fibers/100 μm^2^)* |  |
| Age (cont.) | -7.9×10^-3^ (-0.02, 5.5×10^-3^) |
| BMI (cont.) | -3.4×10^-4^ (-0.01, 0.01) |
| BMI (cat., trend) | -0.03 (-0.12, 0.06) |
| Menopausal status (no/yes) | 9×10^-3^ (-0.16, 0.18) |
| Age at menarche (cat., trend) | -0.08 (-0.17, 5.6×10^-3^) |
| Parity (no/yes) | -0.09 (-0.28, 0.1) |
| Age at first birth (cat., trend) | -0.06 (-0.2, 0.07) |
| Smoking (never/ever) | 0.14 (-0.02, 0.3) |
| Family history (no/yes) | -0.04 (-0.22, 0.13) |
| Prior breast biopsy (no/yes) | 0.02 (-0.15, 0.18) |
| *Median orientation (°)* |  |
| Age (cont.) | 0.02 (-0.16, 0.19) |
| BMI (cont.) | 3.7×10^-3^ (-0.12, 0.13) |
| BMI (cat., trend) | 0.07 (-1.06, 1.2) |
| Menopausal status (no/yes) | 0.13 (-1.71, 1.97) |
| Age at menarche (cat., trend) | 1.04 (-0.15, 2.23) |
| Parity (no/yes) | 0.82 (-0.89, 2.53) |
| Age at first birth (cat., trend) | 0.39 (-0.99, 1.77) |
| Smoking (never/ever) | -0.46 (-2.28, 1.36) |
| Family history (no/yes) | 0.73 (-1.44, 2.9) |
| Prior breast biopsy (no/yes) | -1.6 (-3.37, 0.17) |

^*^Controls were women diagnosed with benign breast disease.

^§^Based on generalized estimating equation (GEE) models with independent correlation structure.

Outcomes of the models were the fiber characteristics. Woman characteristics were the independent variables of the models.

**Table S5.** Associations between mammographic density measures and histologic tissue composition characteristics.

|  | All subjects  (138 women)  β (95% CI)^§^ | Controls^*^  (73 women)  β (95% CI)^§^ | Cases^*^  (65 women)  β (95% CI)^§^ |
| --- | --- | --- | --- |
| *Percent stroma on slide* |  |  |  |
| Global mammographic density |  |  |  |
| % FGV | **0.02 (0.01, 0.04)** | **0.02 (6.3×10^-3^, 0.04)** | **0.03 (8.3×10^-3^, 0.04)** |
| Dense volume | 9.8e-03 (-0.02, 0.04) | -1.4e-03 (-0.04, 0.04) | 0.02 (-0.01, 0.06) |
| Local mammographic density of the biopsy target |  |  |  |
| % FGV | **0.04 (0.02, 0.05)** | **0.04 (0.02, 0.06)** | **0.03 (0.02, 0.05)** |
| Dense volume | **0.02 (7.9×10^-3^, 0.02)** | **0.02 (6.8×10^-3^, 0.03)** | **0.01 (1.8×10^-3^, 0.02)** |
| Volume | **0.01 (2.4×10^-4^, 0.02)** | **0.02 (6.1×10^-4^, 0.03)** | 7.6×10^-3^ (-0.01, 0.03) |
| Radius lesion | **2.3e-03 (6×10^-4^, 4×10^-3^)** | **2.9×10^-3^ (6.8×10^-4^, 5.2×10^-3^)** | 1.5×10^-3^ (-1.1×10^-3^, 4.1×10^-3^) |
| Local mammographic density at 2mm ring |  |  |  |
| % FGV | **8×10^-3^ (4.9×10^-3^, 0.01)** | **9×10^-3^ (4.5×10^-3^, 0.01)** | **6.8×10^-3^ (2.6×10^-3^, 0.01)** |
| Dense volume | 2.7×10^-3^ (-2×10^-3^, 7.4×10^-3^) | 3.7×10^-3^ (-2.4×10^-3^, 9.9×10^-3^) | 1.5×10^-3^ (-5.9×10^-3^, 8.9×10^-3^) |
| Volume | **8×10^-3^ (4.9×10^-3^, 0.01)** | **9×10^-3^ (4.5×10^-3^, 0.01)** | **6.8×10^-3^ (2.6×10^-3^, 0.01)** |
| *Percent epithelium on slide* |  |  |  |
| Global mammographic density |  |  |  |
| % FGV | 9.3×10^-3^ (-0.03, 0.05) | 1.9×10^-4^ (-0.13, 0.13) | 0.01 (-0.03, 0.05) |
| Dense volume | 2.9×10^-4^ (-0.08, 0.08) | -0.17 (-0.41, 0.07) | 0.02 (-0.06, 0.1) |
| Local mammographic density of the biopsy target |  |  |  |
| % FGV | 0.02 (-0.03, 0.06) | 0.04 (-0.09, 0.18) | 0.01 (-0.03, 0.05) |
| Dense volume | 0.02 (-7.1e-03, 0.04) | 0.07 (-9e-03, 0.14) | 0.01 (-0.01, 0.03) |
| Volume | 0.02 (-0.01, 0.06) | 0.08 (-0.02, 0.18) | 0.02 (-0.02, 0.06) |
| Radius lesion | 2.9×10^-3^ (-2.1×10^-3^, 7.9×10^-3^) | **0.01 (2.8×10^-5^, 0.03)** | 1.4×10^-3^ (-3.9×10^-3^, 6.8×10^-3^) |
| Local mammographic density at 2mm ring |  |  |  |
| % FGV | 4.5×10^-3^ (-0.04, 0.05) | 0.03 (-0.11, 0.17) | 10^-3^ (-0.04, 0.04) |
| Dense volume | 3.7×10^-3^ (-6×10^-3^, 0.01) | 0.03 (-6.3×10^-3^, 0.06) | 9.7×10^-4^ (-8.3×10^-3^, 0.01) |
| Volume | 8.1×10^-3^ (-5.5×10^-3^, 0.02) | 0.03 (-0.01, 0.07) | 5.9e-03 (-9.3×10^-3^, 0.02) |
| *Percent fat on slide* |  |  |  |
| Global mammographic density |  |  |  |
| % FGV | **-0.02 (-0.03, -7.4×10^-3^)** | **-0.02 (-0.03, -2.3×10^-3^)** | **-0.02 (-0.03, -3.6×10^-3^)** |
| Dense volume | -7.1×10^-3^ (-0.03, 0.01) | 2.8e-03 (-0.03, 0.04) | -0.02 (-0.05, 0.01) |
| Local mammographic density of the biopsy target |  |  |  |
| % FGV | **-0.03 (-0.04, -0.02)** | **-0.03 (-0.05, -0.01)** | **-0.02 (-0.04, -10^-2^)** |
| Dense volume | **-0.01 (-0.02, -6.5×10^-3^)** | **-0.01 (-0.02, -5.5×10^-3^)** | **-0.01 (-0.02, -1.9×10^-3^)** |
| Volume | **-0.01 (-0.02, -1.1×10^-3^)** | **-0.01 (-0.03, -5×10^-4^)** | -8.4×10^-3^ (-0.02, 7.1×10^-3^) |
| Radius lesion | **-1.9×10^-3^ (-3.3×10^-3^,**  **-4.9×10^-4^)** | **-2.4×10^-3^ (-4.3×10^-3^,**  **-4.5×10^-4^)** | -1.4×10^-3^ (-3.5×10^-3^, 7.3×10^-4^) |
| Local mammographic density at 2mm ring |  |  |  |
| % FGV | **-0.02 (-0.04, -0.01)** | **-0.03 (-0.05, -0.01)** | **-0.02 (-0.04, -5.5×10^-3^)** |
| Dense volume | **-6.2×10^-3^ (-8.8×10^-3^,**  **-3.6×10^-3^)** | **-7.4×10^-3^ (-0.01, -3.5×10^-3^)** | **-4.8×10^-3^ (-8.3×10^-3^, -1.4×10^-3^)** |
| Volume | -2.7×10^-3^ (-6.7×10^-3^, 1.2×10^-3^) | -3.5×10^-3^ (-8.7×10^-3^, 1.7×10^-3^) | -2×10^-3^ (-8×10^-3^, 4.1×10^-3^) |

MD: Mammographic density; FGV: Fibroglandular volume.

^*^Cases were women who received a biopsy diagnosis of either *in situ* (n=32) or invasive (n=33) cancer. Controls were women diagnosed with benign breast disease.

^§^Based on linear models. Outcomes of the models were the square root of the MD. Tissue composition variables were the independent variables of the models.

**Table S6.** Associations between collagen fiber characteristics and tumor characteristics (restricted to cancer ROIs).

|  | Grade  (III *vs* I, II) | | Tumor size  (≥1cm *vs* <1cm) | | ER status  (- *vs* +) | | PR status  (- *vs* +) | |
| --- | --- | --- | --- | --- | --- | --- | --- | --- |
|  | OR (95% CI) | Pval | OR (95% CI) | Pval | OR (95% CI) | Pval | OR (95% CI) | Pval |
| Average length (μm) | 1.16 (0.70, 1.97) | 0.563 | 1.82 (0.90, 4.14) | 0.113 | 0.92 (0.52, 1.61) | 0.773 | 0.93 (0.53, 1.61) | 0.801 |
| Median straightness | **1.80 (1.06, 3.35)** | **0.042** | 1.00 (0.51, 2.00) | 0.994 | 0.90 (0.51, 1.55) | 0.698 | 0.95 (0.55, 1.61) | 0.844 |
| Average width (μm) | 1.84 (0.98, 3.90) | 0.078 | 1.07 (0.51, 2.29) | 0.851 | 0.90 (0.46, 1.68) | 0.746 | 0.95 (0.50, 1.74) | 0.867 |
| Median alignment  (in 44.91μm×44.91μm) | 0.84 (0.45, 1.56) | 0.580 | 1.95 (0.89, 5.09) | 0.115 | 0.78 (0.38, 1.53) | 0.489 | 0.80 (0.39, 1.52) | 0.503 |
| Density (# of fibers/100 μm^2^) | 0.93 (0.46, 1.80) | 0.826 | **3.25 (1.19, 12.01)** | **0.042** | 1.35 (0.65, 3.03) | 0.438 | 0.89 (0.44, 1.81) | 0.742 |
| Median orientation (°) | 0.49 (0.20, 1.00) | 0.071 | 1.07 (0.19, 6.72) | 0.939 | 1.29 (0.62, 2.77) | 0.488 | 1.10 (0.53, 2.22) | 0.793 |

Based on logistic regression models. Outcomes of the models were the tumor characteristics. Fiber characteristics were the independent variables of the models. OR is per 1-standard deviation of the fiber characteristic.
